# Review on Main Gate Characteristics of P-Type GaN Gate High-Electron-Mobility Transistors

**DOI:** 10.3390/mi15010080

**Published:** 2023-12-30

**Authors:** Zhongxu Wang, Jiao Nan, Zhiwen Tian, Pei Liu, Yinhe Wu, Jincheng Zhang

**Affiliations:** 1 Key Laboratory for Wide Bandgap Semiconductor Materials and Devices, School of Microelectronics, Xidian University, Xi’an 710071, China; wzx7240@163.com (Z.W.); 21111212778@stu.xidian.edu.cn (J.N.); 2 China Astronautics Standards Institute, Beijing 100071, China; tianzhiwen708@163.com (Z.T.); liupei_83@163.com (P.L.); 3 Guangzhou Wide Bandgap Semiconductor Innovation Center, Guangzhou Institute of Technology, Xidian University, Guangzhou 510555, China

**Keywords:** gallium nitride, threshold voltage, gate-breakdown voltage, breakdown field, p-type doped gallium nitride high-electron-mobility transistor (p-GaN HEMT)

## Abstract

As wide bandgap semiconductors, gallium nitride (GaN) lateral high-electron-mobility transistors (HEMTs) possess high breakdown voltage, low resistance and high frequency performance. PGaN gate HEMTs are promising candidates for high-voltage, high-power applications due to the normally off operation and robust gate reliability. However, the threshold and gate-breakdown voltages are relatively low compared with Si-based and SiC-based power MOSFETs. The epitaxial layers and device structures were optimized to enhance the main characteristics of pGaN HEMTs. In this work, various methods to improve threshold and gate-breakdown voltages are presented, such as the top-layer optimization of the pGaN cap, hole-concentration enhancement, the low-work-function gate electrode, and the MIS-type pGaN gate. The discussion of the main gate characteristic enhancement of p-type GaN gate HEMTs would accelerate the development of GaN power electronics to some extent.

## 1. Introduction

As power electronic products continue to prosper in diverse fields and industries, gallium nitride (GaN)-based high-electron-mobility transistors (HEMTs) have been developing rapidly. In contrast to traditional silicon-based devices, the rationale of gallium nitride-based HEMT devices relies upon the polarization effect of GaN materials, which accumulates two-dimensional electron gas (2DEG) on the AlGaN/GaN heterojunction area to form lateral conductive channels for electric conduction. Owing to the polarization characteristics of gallium nitride material, conventional GaN HEMT devices are depletion-mode electronics with negative threshold voltage [1,2,3,4,5,6,7]. In practical applications, depletion-mode devices require a negative-voltage power supply, which increases not only the complexity of the circuit but also the costs. In addition, certain safety hazards may happen to normally on devices, so the electronics industry urgently demands enhanced-mode devices rather than depletion-mode ones.

Currently, the most commonly used methods for fabricating enhanced-mode GaN-based HEMT devices primarily include adopting a grooved-gate structure, implanting a fluorine ion, and inserting p-type doped GaN cap layers, as well as applying a cascode structure. Among the above prevailing methods, the most reliable and widely adopted one is to use p-GaN as the gate cap layer to improve the threshold voltage of the device.

Unfortunately, the p-GaN gate enhanced-mode HEMT devices are still invariably confronted with two critical problems of low threshold voltage (V_TH_) (approximate +1 V) and low gate-breakdown voltage (below 10 V). Specifically, the former may induce erroneous triggering of the device, whereas the latter is prone to cause gate breakdown and/or even burnout of the device. In response to these problems, it is of vital importance to develop a p-GaN HEMT device characterized by a high threshold voltage and a high gate in order to improve the reliability and robustness of the device in circuit applications while improving its efficiency and reducing its power consumption.

This paper mainly reviews research progress in improving the threshold voltage and gate-breakdown voltage of p-GaN HEMT devices in recent years. This paper is organized as follows. Section 2 introduces the existing problems of the above types of devices, such as low threshold voltage, low gate-breakdown voltage, etc. Section 3 formulates the structures of traditional p-GaN HEMT devices, of high-threshold devices, and of high gate-breakdown voltage ones. Section 4 summarizes the paper and proposes a future research focus.

## 2. Problems to Be Solved

### 2.1. Low Threshold Voltage

The fabrication of a pGaN HEMT device mainly adopts Mg^2+^ doping to realize normally off operation, where the 2DEG exists within the channel. Specifically, a higher doping concentration of Mg^2+^ ions realizes a higher threshold voltage of the device. Conversely, if the doping concentration of the Mg^2+^ ions is too low, its depletion effect imposed on the 2DEG will be limited, which thus lowers the threshold voltage of the device. In practical situations, since the acceptor activation energy of Mg^2+^ is high, it is difficult to activate it. As a result, increasing the activation rate of p-type doping in GaN still remains difficult, which further results in the low threshold voltage of p-type gate devices [8].

### 2.2. Low Gate-Breakdown Voltage

The gate metal of p-GaN HEMT can form ohmic or Schottky contact with p-GaN. The gate structure with ohmic contact has a large amount of forward leakage and small gate swing. Aiming to reduce gate leakage while increasing gate swing, Schottky contact predominates currently in gate structures [9,10].

Due to the inherent gate structure of Schottky contact, the gate-breakdown voltage of a GaN HEMT device characterized by Schottky contact is low. This structure has two back-to-back diodes. With the increase in the gate bias, a depletion region would be formed, and gate breakdown can occur for a large gate bias. Table 1 lists the threshold voltages of the prevailing p-GaN power devices on the market, which range from 1.0 V to 1.6 V; the gate-breakdown voltage is no higher than 7 V [11]. In contrast, the threshold voltages of Si- and SiC-based metal-oxide-semiconductor field-effect transistor (MOSFET) devices range from 2.5 V to 6 V, and the gate-breakdown voltages exceed 18 V, which is much higher than those of p-GaN-based HEMT devices, as shown in Table 2. This suggests that the conduction resistance of a MOSFET can be efficiently reduced by increasing the gate-driving voltage [12].

However, the above approach is not applicable to p-GaN devices. The reason is that the safety margin of the gate-drive voltage is approximately 4~5 V, whereas the threshold voltage and gate-breakdown voltage of this type of device are relatively low. If the threshold voltage is low, electromagnetic interference may cause the gate electrode to open incorrectly. On the other hand, the inductance of a high-frequency driving circuit may cause driving-voltage oscillation, under which circumstance the gate voltage may exceed the breakdown limit. This will further result in gate degradation and a more complicated driving circuit, aggravating the circuit power consumption [13].

## 3. Research Progress

### 3.1. Structure of Traditional p-GaN HEMT

**Figure 1:**
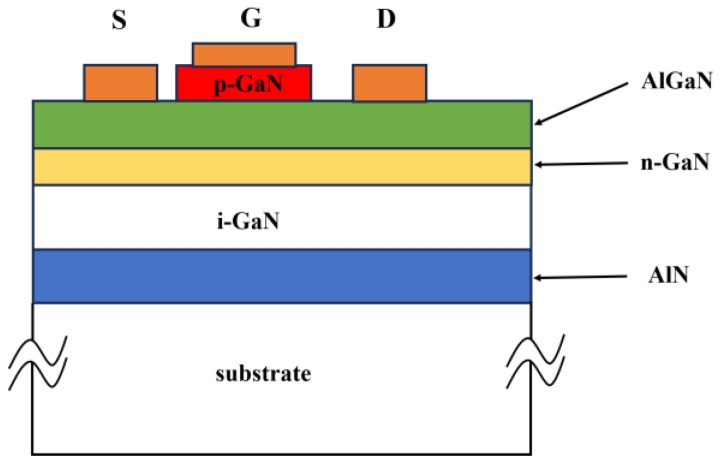
Structural diagram of p-GaN HEMT device. Adapted from Ref. [14].

By using a selective growth method, Hu et al. inserted a p-type doped gallium nitride layer between the AlGaN/GaN heterojunction material and the gate of the HEMT device [14,15], as shown in Figure 1. This method depletes the 2DEG existing in the channel of the device by enhancing the energy band of the AlGaN barrier through utilizing p-GaN, thereby realizing enhancement-mode HEMT for the first time ever recorded by adopting a p–n junction. Although the threshold voltage of the fabricated device is less than 1 V and its saturation current is only 40 mA/mm, it offers an idea for the future development of p-GaN HEMT.

**Figure 2:**
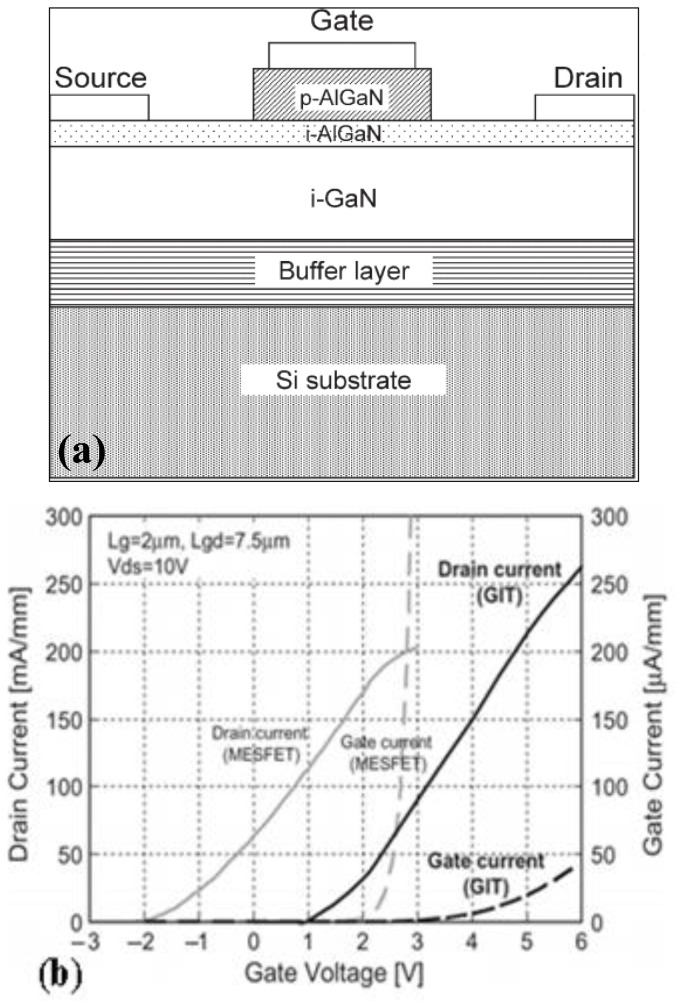
Traditional p-AlGaN HEMT device and its (**a**) structural diagram and (**b**) transfer characteristic curve [16]. Reprinted/adapted with permission from Ref. [16]. 2007, IEEE.

Subsequently, Uemoto et al. used p-AlGaN as the gate material to grow a layer of p-AlGaN on the AlGaN barrier. The inductively coupled plasma (ICP) etching technology was then used to remove the p-AlGaN that existed between the gate–source bridge and the gate drain, during which the p-AlGaN of the gate was used to exhaust the 2DEG [16,17], as shown in Figure 2. This approach utilizes a hole injection effect to realize enhancement-mode characteristics of the device, so this type of device is also known as a gate injection transistor (GIT). At the gate voltage of 0 V, the channel under the gate is fully depleted, and the drain current does not flow. At the gate voltages up to the forward built-in voltage of the p–n junction, the GIT is operated as a field-effect transistor. A further increase in the gate voltage exceeds the results of the hole injection to the channel from the p-AlGaN. The injected holes accumulate an equal number of electrons that flow from the source to keep charge neutrality at the channel. The accumulated electrons are moved by the drain bias with high mobility, while the injected holes stay around the gate because the hole mobility is at least two orders of magnitude lower than that of the electron. This conductivity modulation results in a significant increase in the drain current.

The threshold voltage of the above as-prepared device achieved +1 V threshold voltage, its maximum gate-breakdown voltage was 6 V, and the saturation output current was 200 mA/mm. However, due to the low activation concentration of p-AlGaN material, the threshold voltage of the device remained low.

**Figure 3:**
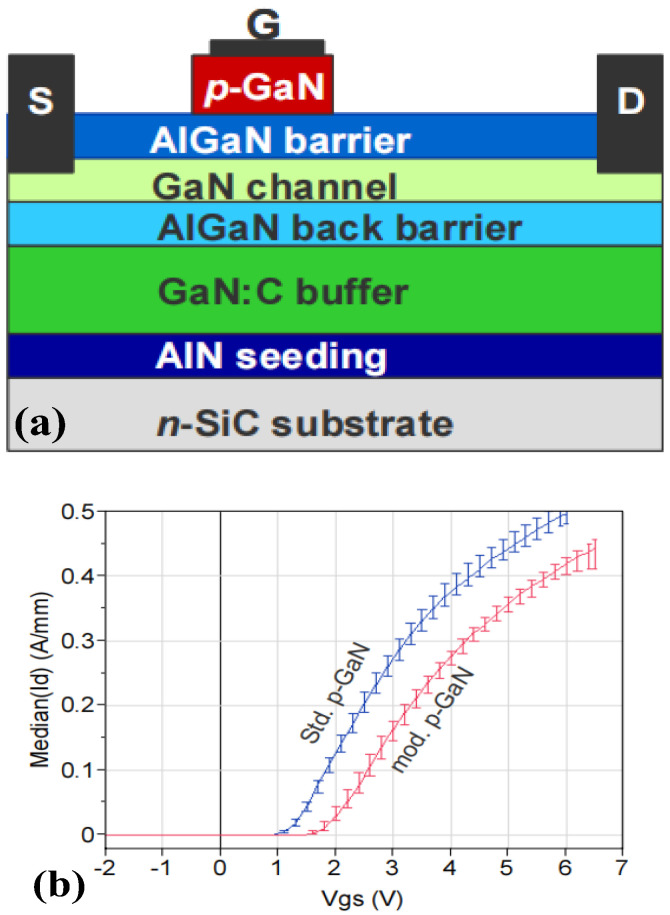
(**a**) Structural diagram and (**b**) transfer characteristic curve of the p-GaN HEMT device with back barrier [18]. Reprinted/adapted with permission from Ref. [18]. 2011, IEEE.

In order to realize an enhancement-mode p-GaN HEMT device while increasing its breakdown voltage, Hilt et al. combined the inserted p-GaN layer with the AlGaN back barrier, adopting AlGaN as the epitaxial layer to function as the back barrier to suppress the penetration current between the source and drain in the off-state [18,19], as shown in Figure 3. The fabricated device achieved 1.78 V threshold voltage and 350 mA/mm saturation output current, and its off-state breakdown voltage exceeded 1000 V. The used GaN:C-buffer in combination with the AlGaN back barrier gave a very high breakdown strength to the devices that scaled with 170 V/µm gate-drain distance. Since the device can be categorized as a conventional p-GaN in terms of structure, its threshold voltage is not high enough, its gate-breakdown voltage is relatively low, and a large gate-leakage current still exists.

**Figure 4:**
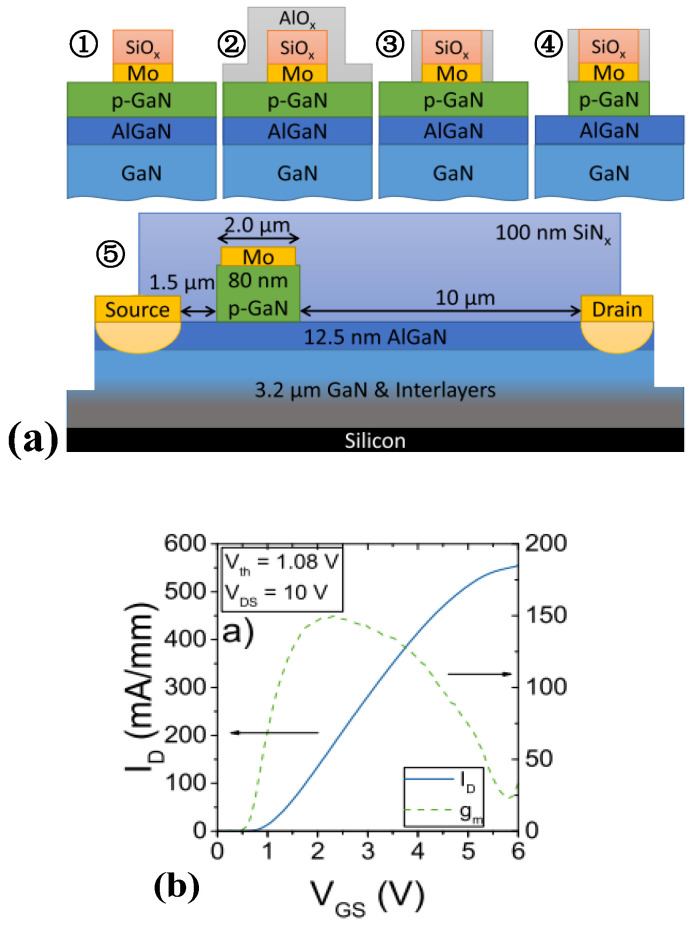
(**a**) Structural diagram and (**b**) transfer characteristic curve of p-GaN HEMT device adopting gate-first process [20]. Reprinted/adapted with permission from Ref. [20]. 2018, IEEE.

With respect to optimizing the preparation process of p-GaN HEMT devices, Lukens et al. adopted the gate-first process, as shown in Figure 4. This process is also known as the metal-implanted polycrystalline silicon (MIPS) gate process, which refers to implanting a layer of the high-melting-point metal TiN and different work function layers between a high K medium and a polysilicon gate. The notion of the work function layer is also known as the covering layer, and the purpose of implanting TiN is to solve the gate-depletion phenomenon in the MIPS gate process; the work function covering layer is capable of mitigating the degree of Fermi-level pinning.

Specifically, this approach first evaporates the gate metal, while the metal layer can perform as an etching mask, thereby achieving a self-aligned process and improving etching precision [20,21]. The self-aligned gate metallization has been realized by an encapsulated Mo gate, while selective p-GaN etching has been established by a Cl_2_/N_2_/O_2_ gas mixture in an ICP-RIE plasma etching tool. Selective etching and self-alignment have allowed for p-GaN-gated HFET processing with very good etch depth control and excellent electrical properties. By protecting the gate region of the device, the above approach achieves a threshold voltage of 1.08 V and a saturation output current of 554 mA/mm of the prepared device. Nevertheless, the device still belongs to conventional electronics in terms of structure, exhibiting a low threshold voltage and a high gate-leakage current.

### 3.2. Structure of p-GaN HEMT Device with High Threshold Voltage

**Figure 5:**
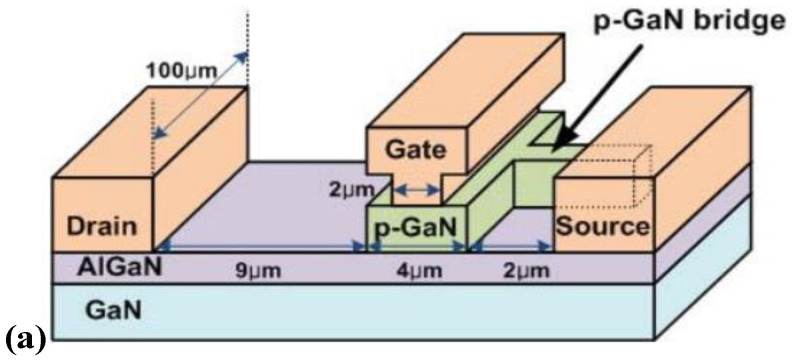

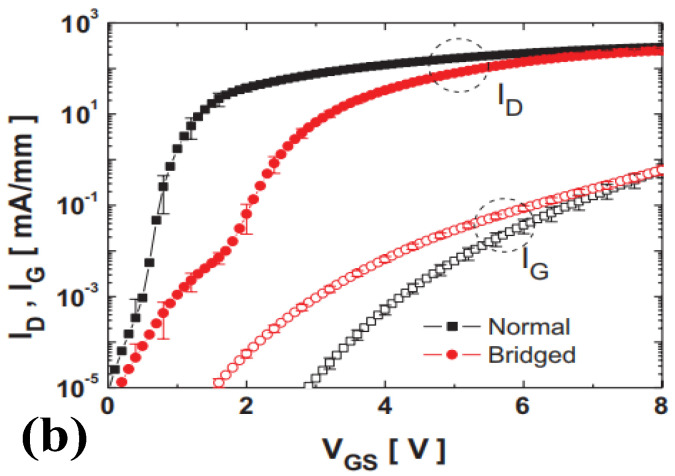
(**a**) Structural diagram and (**b**) transfer characteristic curve of p-GaN HEMT device with gate–source bridge [22]. Reprinted/adapted with permission from Ref. [22]. 2013, IEEE.

Aiming to increase the threshold voltage of p-GaN HEMT devices, various novel device structures have been proposed, among which the structure of the gate–source bridge was first proposed by Hwang et al. [22,23]. The above method bridges the gate and source electrodes, by which the width of the depletion region can be increased, thereby improving the threshold voltage of the device, as shown in Figure 5. When the width of the bridge increases, the threshold voltage increases because the resistance of the bridge and the source-to-p-GaN bridge contact decreases. However, there is a tradeoff, since an increase in bridge width reduces the 2DEG channel area, which increases resistance. By adopting this structure, the threshold voltage of p-GaN gate HEMT devices can be increased from 0.93 V to 2.44 V. However, it must be noted that, as the bridge width increases, the specific on-resistance of the device also increases, whereas its peak transconductance decreases.

**Figure 6:**
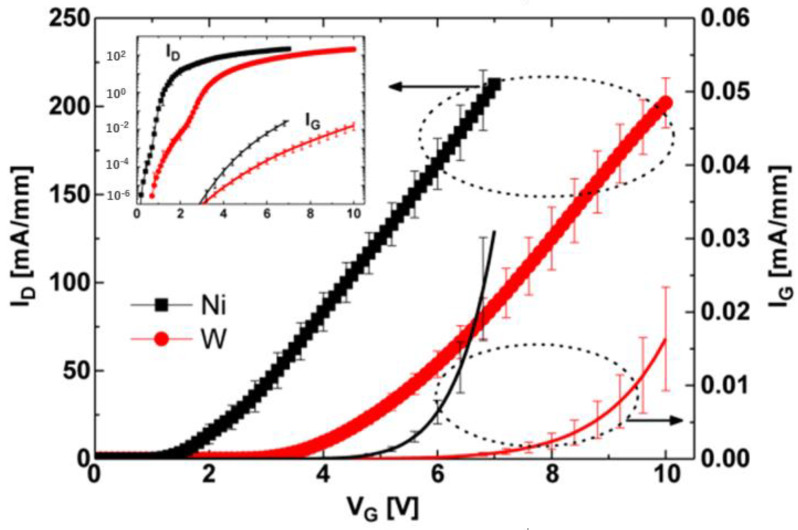
Transfer characteristic curve of p-GaN HEMT device with tungsten gate [24]. Reprinted/adapted with permission from Ref. [24]. 2013, IEEE.

One alternative that is capable of effectively increasing the threshold voltage of a p-GaN HEMT device is adopting a low-power-function layer to increase the Schottky barrier of the device. Hwang et al. used magnetron-sputtered tungsten instead of nickel to function as the gate metal of a p-GaN HEMT device [24,25]. This is because tungsten has a lower metal work function and can form a higher Schottky barrier with p-GaN, as shown in Figure 6. A Schottky contact between a gate metal and p-GaN generates a hole depletion region at the interface, and the width of the depleted layer increases as the work function of the gate metal decreases. As the gate bias increases, the contact of the p-GaN with a low-work-function metal shows an increased depletion width, whereas the contact with a high-work-function metal shows no change in the depletion width in p-GaN, similar to typical ohmic contact behavior. Therefore, in the case of a gate metal with a low work function, the gate bias is partially applied in the p-GaN due to the increased depletion width in the p-GaN. By using this method, the threshold voltage of the as-prepared device was increased from 1.2 V to 3 V, and its saturation output current reached 260 mA/mm.

**Figure 7:**
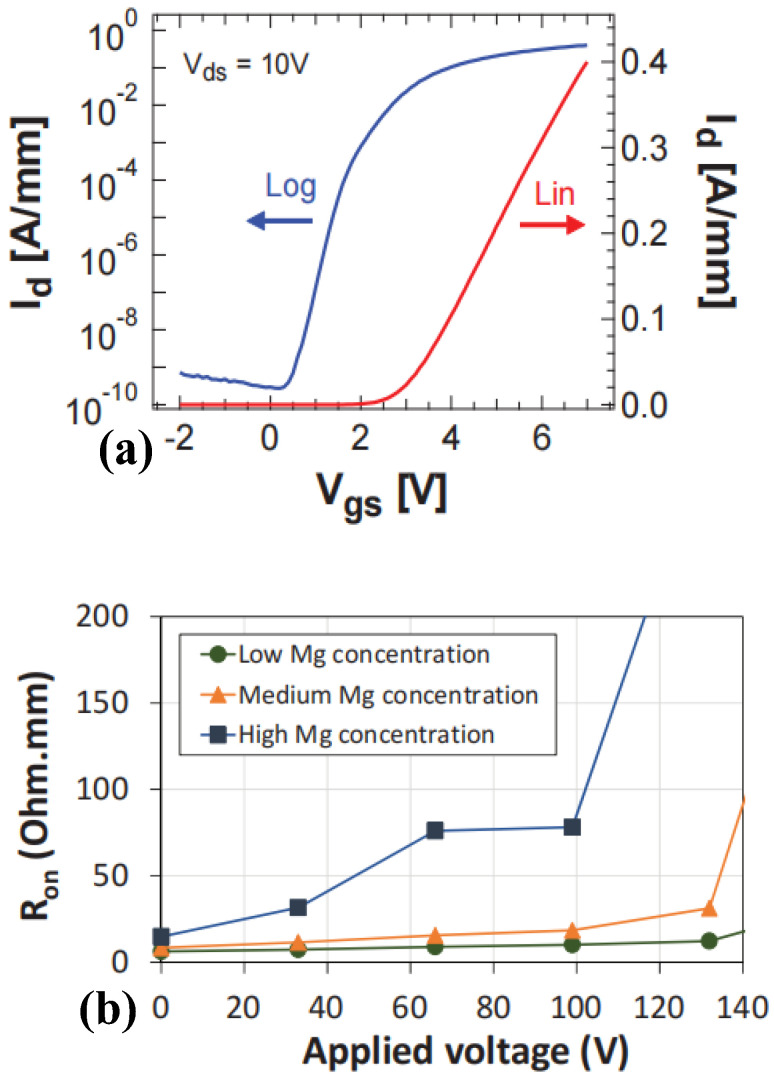
p-GaN HEMT device and (**a**) its transfer characteristic curve and (**b**) the curve of specific on-resistance varying with Mg^2+^ concentration [8]. Reprinted/adapted with permission from Ref. [8]. 2016, IEEE.

In response to the difficulty of improving the activation rate of Mg^2+^ in p-GaN, Posthuma et al. investigated the effects of Mg^2+^ diffusion and activation in p-GaN layers imposed on the primary performance of HEMT devices [8]. By optimizing the activation temperature of Mg^2+^ and the growth temperature of the p-GaN layer, the effect of Mg^2+^ diffusion on 2DEG is therefore reduced while increasing the activation concentration of Mg^2+^. This approach increases the threshold voltage of the device while ensuring the on-state resistance of the device. As shown in Figure 7, the threshold voltage and the specific on-resistance of the as-prepared, enhancement-mode HEMT device are 2.1 V and 150 mΩ, respectively.

**Figure 8:**
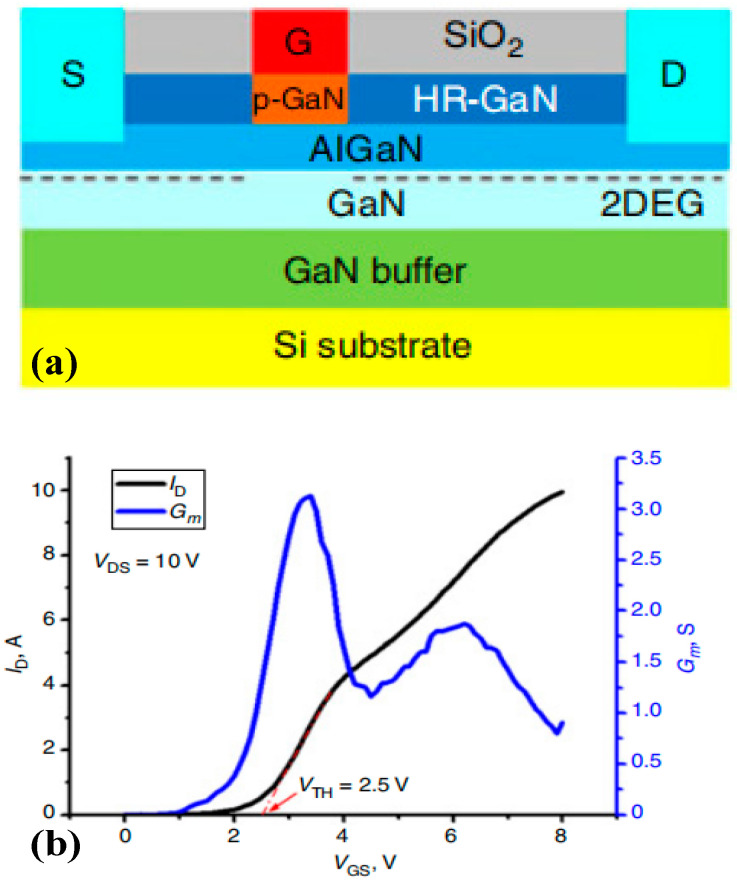
p-GaN HEMT device (**a**) adopting hydrogen plasma processing and (**b**) its transfer characteristic curve [26]. Reprinted/adapted with permission from Ref. [26]. 2018, IEEE.

Among diverse technological difficulties in manufacturing p-GaN HEMT devices, a critical one is precise etching. To address this problem, Hao et al. adopted hydrogen plasma-processing technology [26]; the rationale of this approach is shown in Figure 8. A self-alignment hydrogen plasma treatment was adopted to passivate the p-GaN cap layer in the access region. Due to the hole compensation effect generated by hydrogen atoms that exist in p-type Mg-doped GaN to form a Mg-H neutral complex, the Mg receptor is therefore passivated in the p-type GaN layer, which transforms the p-GaN layer outside the gate into high-resistance GaN, thereby omitting the process of ICP etching. By using this method, the fabricated device exhibits normally off operation with a high-threshold voltage of 2.5 V, a maximum drain current of 10 A, a breakdown voltage of 567 V, and a low forward gate-leakage current of 1.3 × 10^−7^ A/mm at V_GS_ = 8 V.

**Figure 9:**
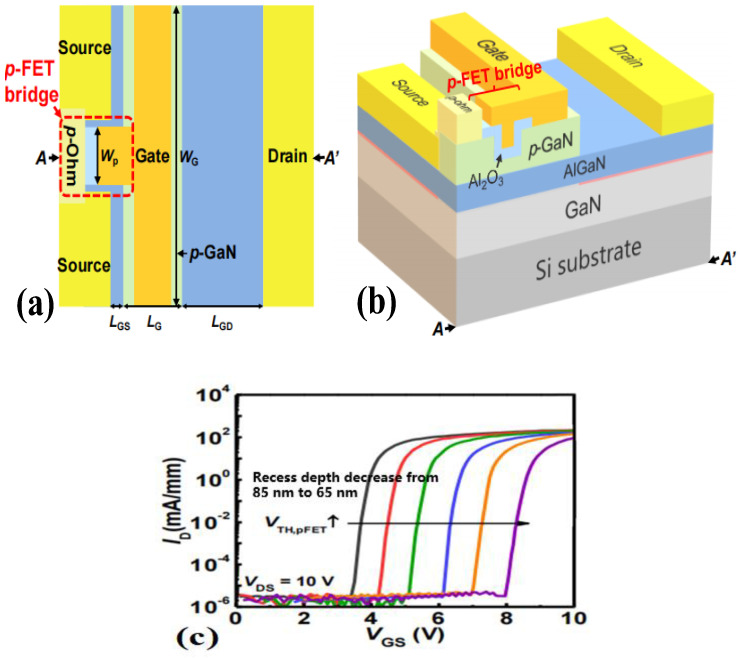
Gate–source bridge p-GaN HEMT device and (**a**) its vertical view, (**b**) three-dimensional structural diagram, and (**c**) transfer characteristic curves of p-FET bridges with different depths of recessed gate [27]. Reprinted/adapted with permission from Ref. [27]. 2020, IEEE.

Based upon the existing research on the gate–source bridge, Hua et al. introduced a gate–source bridge between the gate and the source of p-GaN HEMT [27], and adopted a p-type ohmic contact near the source and a p-type bridge, an oxide layer below the gate–source bridge also being introduced. The metal–insulator–semiconductor (MIS) structure formed by the oxide layer with metal and with p-GaN can divide the voltage, while the lower trap state on the surface of p-GaN can effectively reduce the gate-leakage current. As shown in Figure 9, by adjusting the p-type doped field-effect transistor (p-FET) bridge with different depths of the recessed gate, the threshold voltage of the fabricated device is increased from 3.6 V to 8.2 V.

### 3.3. Structure of p-GaN HEMT with High Gate-Breakdown Voltage

**Figure 10:**
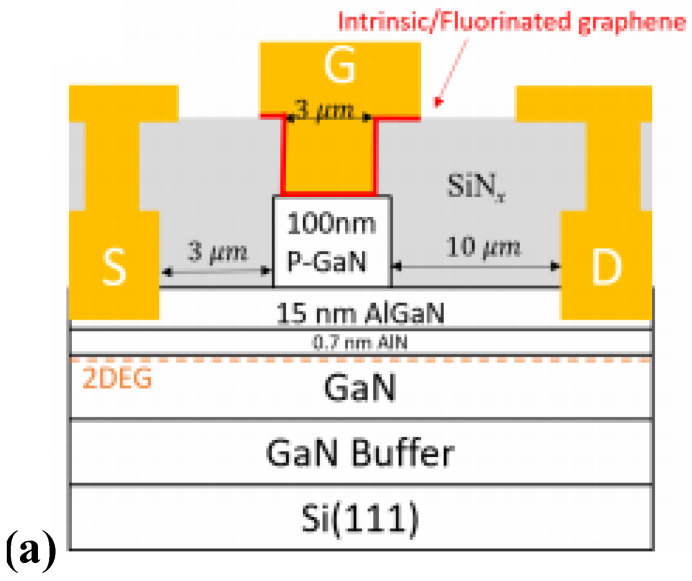

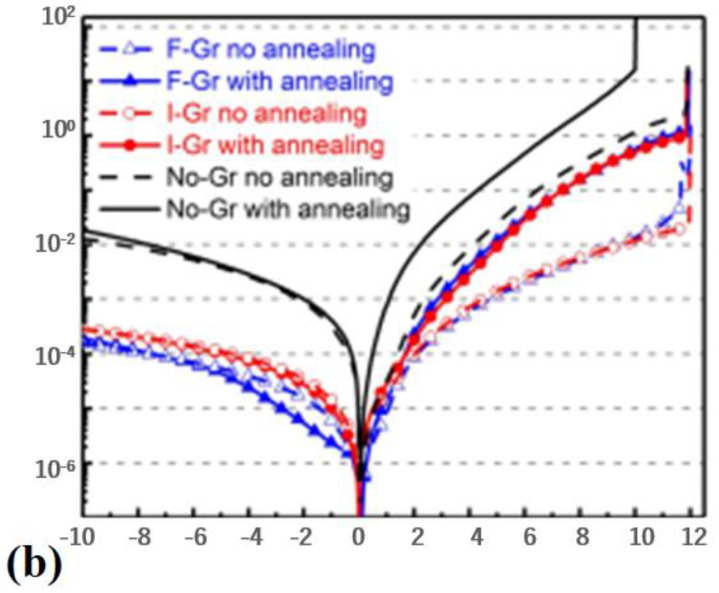
P-GaN HEMT device with fluorographene and titanium fabricated gate and its (**a**) structural diagram and (**b**) characteristic curves demonstrating gate-breakdown voltage and gate-leakage current [29]. Reprinted/adapted with permission from Ref. [29]. 2019, IEEE.

Regarding the p-GaN HEMT device with a Schottky gate, one of the most effective methods to improve the gate-breakdown voltage is increasing the height of its Schottky barrier [28]. As shown in Figure 10, Zhou et al. adopted fluorographene and titanium as the metal for fabricating the gate of a p-GaN HEMT device [29]. Different from conventional metal gate devices, fluorographene increases the Schottky barrier height of the fabricated device by 0.24 eV, thereby effectively improving the threshold voltage of the device. Furthermore, the interface quality of the device is improved accordingly, lowering the surface electron density state of p-GaN HEMT effectively. By applying the above technique, the off-state leakage current of the device is reduced by 50 times, and its gate-breakdown voltage achieves 12.1 V, exhibiting excellent temperature stability.

**Figure 11:**
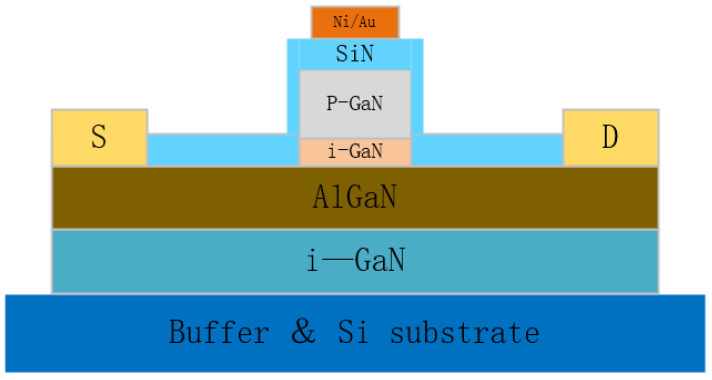
MIS p-GaN HEMT structural diagram. Adapted from Ref. [31].

Another method that is capable of effectively improving the gate voltage of a p-GaN HEMT device is to grow a passivation layer on it. Pu et al. grew a SiN passivation layer on a p-GaN layer [30,31], the structural diagram of which is shown in Figure 11. This approach combines the gate metal, passivation layer, and p-GaN of the device together to form an MIS structure, which greatly reduces the trapped surface charges of the device, thereby mitigating the gate-leakage current. For the SiN–MIS gate structure, a threshold voltage of 2.5 V, gate swing of 16 V, and drain current density of 50 mA/mm were observed. The gate-leakage current was reduced to a very low level in both positive and negative bias regions. The positive shift of V_TH_ for the MIS gate device is ascribed to voltage distribution across the dielectric, while, for the Schottky gate device, there is a relatively lower distance from the conduction band bottom to the Fermi level. 

**Figure 12:**
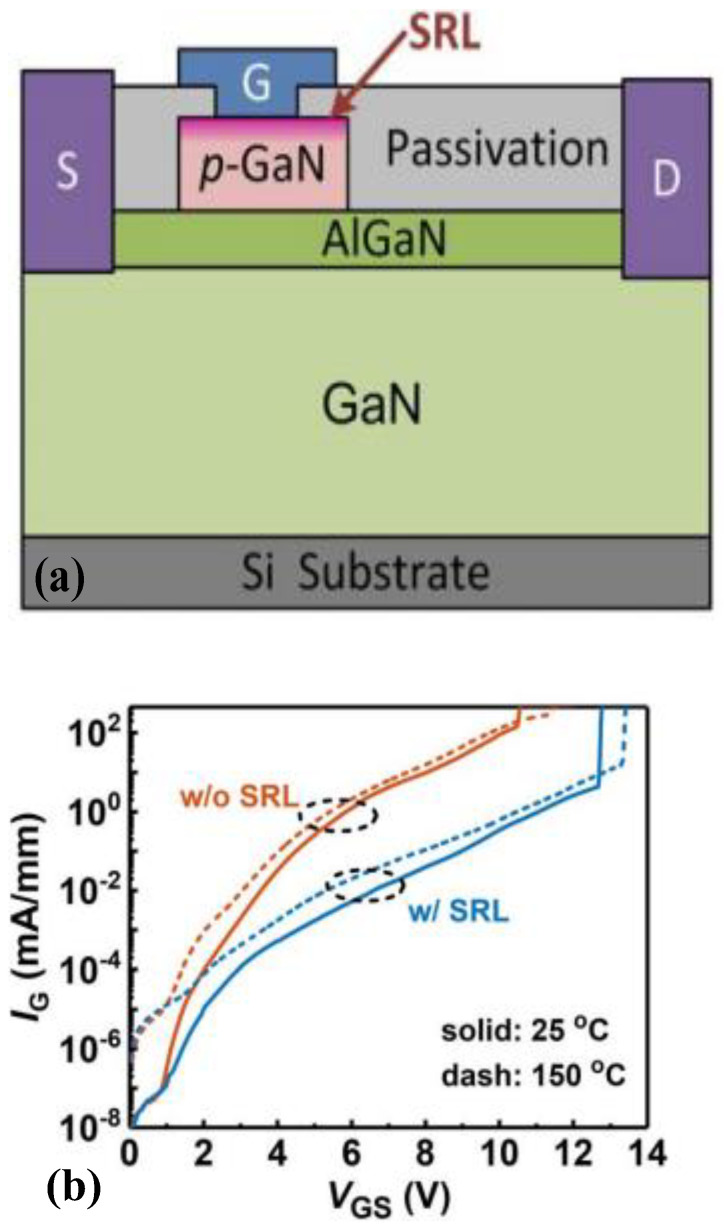
Oxygen plasma-processed p-GaN HEMT device and its (**a**) structural diagram and (**b**) characteristic curves of gate-breakdown voltage [32]. Reprinted/adapted with permission from Ref. [32]. 2021, IEEE.

Research on the surface processing of p-GaN to improve the gate-breakdown voltage has also attracted much attention. Zhang et al. transformed several nanometers of p-GaN near the gate surface into GaON, forming a surface-reinforcement layer (SRL) [32]. Specifically, this approach adopts oxygen plasma processing before depositing metal on the surface of the p-GaN gate, after which the gate is annealed at 800 °C to reconstruct its surface. The surface reconstruction can significantly reduce the trapped surface charges of p-GaN, thereby reducing the gate leakage of the device. In addition, the GaON formed on the gate surface of the device can be approximately equivalent to a very thin passivation layer, which forms a structure similar to MIS, thereby effectively dividing the voltage. It can be observed from Figure 12 that, through adopting this technology, the gate-leakage current of the device is reduced by two orders of magnitude, while the gate-breakdown voltage is increased from 10.5 V to 12.7 V. With the Fowler–Nordheim tunneling fitting of I_G_-V_GS_ curves at high V_GS_, a higher Schottky barrier height of 1.1 eV could be extracted for the device with SRL compared with 0.6 eV of the device without SRL. The higher Schottky barrier height potentially indicates a wider bandgap of GaON. A maximum gate-breakdown voltage of 7.8 V could be obtained for devices with SRL for a 10-year lifetime at a failure level of 1%, which is much higher than the 5.9 V of devices without SRL.

**Figure 13:**
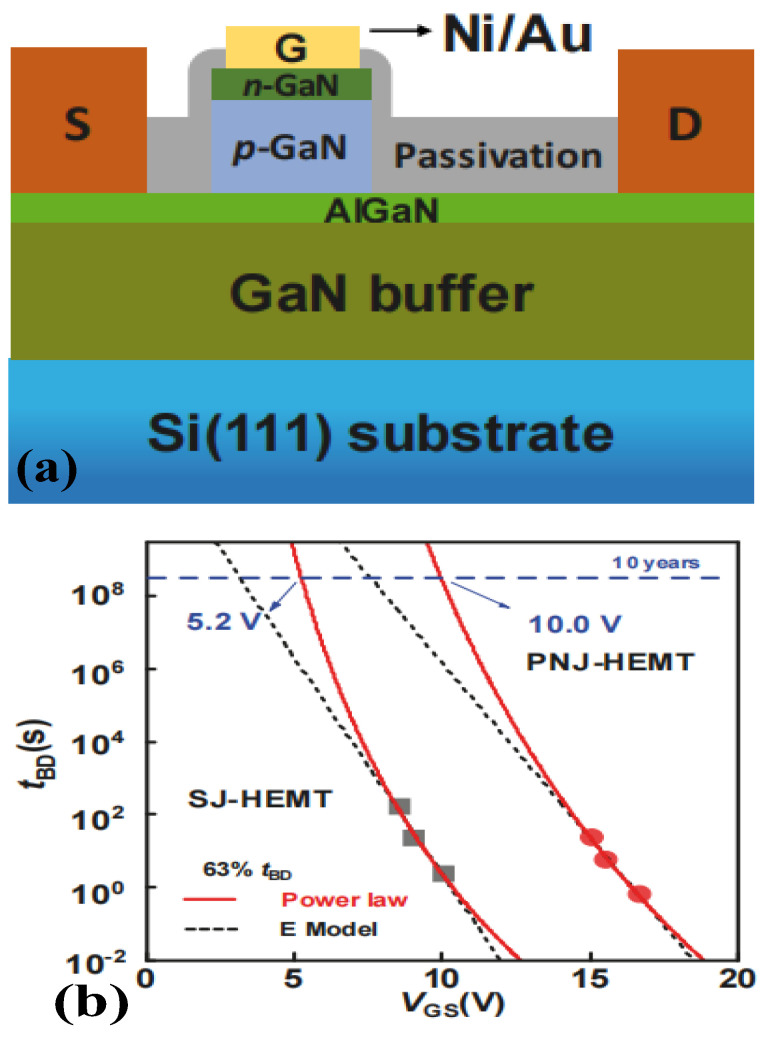
P–n junction HEMT device and its (**a**) structural diagram and (**b**) lifetime prediction of gate-breakdown voltage [33]. Reprinted/adapted with permission from Ref. [33]. 2020, IEEE.

As shown in Figure 13, Wang et al. formed a p–n junction by growing a layer of n-GaN on p-GaN to replace the Schottky junction formed by p-GaN and the gate metal [33]. This approach not only utilizes the reverse bias voltage generated by the p–n junction to increase the gate swing but also reduces the trapped surface charges of the p-GaN device, by which the gate leakage current of the device is thus lowered noticeably. With the same peak E-field, the reverse-biased p–n junction could hold a higher voltage than the Schottky junction, owing to the additional voltage dropped on the n-GaN layer. Compared with the Schottky junction, the holes injected from the gate metal could also be reduced by the p–n junction, because of a higher and thicker barrier under the same forward gate bias. In addition, the peak E-field is buried within the p–n junction and becomes less affected by the surface conditions. At a 63% failure level, the maximum gate-breakdown voltage of the prepared device with a 10-year lifetime is 10 V (power law model), which is much higher than the 5.2 V for the conventional HEMT.

**Figure 14:**
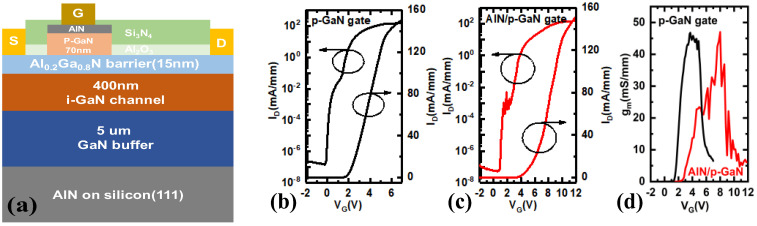
(**a**) Structural diagram and (**c**) transfer curves of in situ AlN/p-GaN gate HEMT device, (**b**) transfer curve of conventional p-GaN gate HEMT, and (**d**) transfer curves comparison [34]. Reprinted/adapted with permission from Ref. [34]. 2023, IEEE.

The in situ-grown p-GaN gate dielectric is also an effective alternative to increase the threshold voltage and gate-breakdown voltage of a p-GaN HEMT device. By in situ growing an AlN dielectric layer on the p-GaN cap layer, Wu et al. improved the reliability of the gate [34]. As shown in Figure 14, the in situ AlN layer is capable of modulating the energy band without introducing trapped surface charges at the AlN/p-GaN interface. Moreover, the in situ AlN layer can also divide the voltage, increasing the gate-breakdown voltage effectively. However, the in situ AlN also has drawbacks. The AlN material’s high stress can easily lead to defects and dislocations, and its quality can be further improved by optimizing its thickness and growth condition.

Compared with a conventional p-GaN HEMT device, the threshold voltage and gate-breakdown voltage of the prepared in situ AlN/p-GaN gate HEMT device are increased from 1.8 V and 10.0 V to 3.9 V and 17.6 V, respectively. At a 63% failure level, the maximum gate-breakdown voltage of the as-prepared device within its 10-year lifespan reached 12.1 V, which is currently the highest level ever reported. Compared with the p-GaN gate HEMT, a part of the gate voltage will drop in the single-crystal AlN dielectric layer, and the gate swing of the AlN/p-GaN gate HEMT will be larger. In situ AlN on the p-GaN cap layer can avoid the introduction of traps at the AlN/p-GaN interface and improve the gate reliability.

## 4. Discussion

GaN E-mode devices include two main structures, namely ohmic-gate and Schottky-gate structures. Compared with ohmic-gate p-GaN HEMTs, p-GaN HEMTs with a Schottky-gate structure possess a higher threshold voltage, a higher gate-breakdown voltage, and a larger gate voltage swing. Although the Schottky-type p-GaN-gate HEMT has yielded forward gate-breakdown voltages larger than 10 V, the maximum gate bias allowed for long-term reliable operation is about 7 V due to gate/p-GaN interface degradation induced by gate leakage under a high electric field. As a result, the gate bias window for complete turn-on of the Schottky-type p-GaN-gate power HEMT is still relatively low, which in turn imposes the need to suppress gate ringing and false turn-on in high-frequency power-switching applications. It is highly desirable to develop device structures that can further reduce the gate leakage and boost the forward gate-breakdown voltage, so that a larger gate drive bias window can be obtained for safe operation. Various methods should be further investigated and optimized, including low work function gate metal, an oxygen plasma-processing layer, an n-GaN cap, and in situ dielectric. In addition, the threshold voltage drift and on-resistance instability are important issues that need to be studied [35,36,37].

## 5. Conclusions

This paper summarizes the research progress into threshold voltage and gate-breakdown voltage in pGaN high-electron-mobility transistors. The threshold voltage of conventional pGaN devices is difficulty to exceed 1.6 V, which makes the device prone to mistakenly turning on during operation. The device’s threshold voltage can be enhanced by methods such as a gate–source bridge, low-work-function tungsten metal, and Mg-doping optimization. The main method to increase the threshold voltage is to deposit a layer of n-type GaN, an oxide layer, a gate dielectric, and other materials on top of the pGaN material. The in situ AlN structure achieves both high threshold voltage and high gate-withstand voltage without introducing additional defects. The commercialization of pGaN gate devices requires reliability assessments such as HTRB and HTGB, besides high threshold voltage and high gate-breakdown voltage. With the development of technology, the threshold voltage and gate-withstand voltage characteristics of pGaN HEMT will continue to improve.

## Figures and Tables

**Table 1:** Parameters of prevailing p-GaN power devices on the market.

Corporate	Model	Voltage Level	Threshold Voltage	Gate-Breakdown Voltage
Efficient Power Conversion	EPC2110	120 V	1.6 V	6 V
GaN Systems	GS66502B	650 V	1.5 V	6 V
STMicroelectronics	SGT120R65AL	650 V	1.0 V	7 V
Innoscience	INN650D140A	650 V	1.5 V	7 V
Sanan IC	SMG060E015L	650 V	1.0 V	6 V

**Table 2:** Parameters of prevailing Si- and SiC-based MOSFET devices.

Material	Corporate	Model	Voltage Level	Threshold Voltage	Gate-Breakdown Voltage
Si	Infineon	IPB65R075CFD7A	650 V	5 V	20 V
	STMicroelectronics	STP10NK60Z	600 V	3.5 V	30 V
SiC	Infineon	IMZA65R048M1H	650 V	6 V	18 V
	Cree	C3M0120065J	650 V	4 V	19 V
	STMicroelectronics	SCT070HU120G3AG	1200 V	4.5 V	18 V

## Data Availability

Not applicable.
